# Prevalence of *Porphyromonas gingivalis* fimbriae A genotypes II and IV in patients with chronic periodontitis and atherosclerosis

**DOI:** 10.15171/japid.2018.009

**Published:** 2018-12-25

**Authors:** Mohammad Reza Talebi Ardakani, Azadeh Esmaeil Nejad, Bahram Kazemi, Seyed Khalil Forouzan Nia, Mohamad Poormohamadi, Haniyeh Moaven, Mohammad Reza Hosseini Kordkheili

**Affiliations:** ^1^Department of Periodontics, Dental School, Shahid Beheshti University of Medical Sciences, Tehran, Iran; ^2^Department of Biotechnology, Medical School, Shahid Beheshti University of Medical Sciences, Tehran, Iran; ^3^Department of Cardiovascular Surgery, Afshar Hospital, Yazd University of Medical science, Yazd, Iran; ^4^Private Practice, Isfahan, Iran; ^5^Private Practice, Tehran, Iran; ^6^Dental Student, Dental School, Shahid Beheshti University of Medical Sciences, Tehran, Iran

**Keywords:** Atherosclerosis, chronic periodontitis, fimbriae, genotypes II and IV, Porphyromonas gingivalis

## Abstract

**Background:**

Atherosclerosis is known as one of the chronic diseases with high prevalence in the human species. Many studies have elucidated the relationship between this disease and chronic periodontitis caused by Porphyromonas gingivalis (P.g). The aim of this study was to investigate the prevalence of P.g fimbriae A (fimA) genotypes II and IV in patients with periodontitis and atherosclerosis.

**Methods:**

This cross-sectional study investigated the frequency of P.g II and IV genotypes in the subgingival plaque specimens of 42 subjects in three experimental groups: periodontitis (A), atherosclerosis (B), periodontitis + atherosclerosis (C) and aortic wall specimens obtained from 30 patients (groups B and C) by the PCR technique.

**Results:**

P.g bacterium was seen in 46.6% of patients with chronic periodontitis. The same bacterium was not found in aortic wall specimens of patients with chronic periodontitis (group C) and there was only one P.g-positive aortic wall specimen (7.7%) among the patients with healthy periodontium (group B). Genotypes II and IV were not observed in any specimen.

**Conclusion:**

The results of statistical analysis showed no significant correlation between the prevalence of P.g and genotypes II and IV in the subgingival plaques and the incidence and severity of atherosclerosis.

## Introduction

Atherosclerosis, a major cause of mortality in industrial societies, is a pathological condition in which lipid plaques are deposited in the inner wall of arteries.^1^ Atherosclerosis accounts for one out of three deaths occurring in the United States.^2^ Many studies have shown the effects of periodontal diseases on elevated atherosclerotic risk in individuals.^3,4^ This can be attributed to different reasons. For example, the initial origins of these diseases might be the same (such as diabetes, smoking of cigarettes or stress).^5,6^ Alternatively, inflammation and periodontal infection might release endotoxins or bacteria in the circulatory system,^7^ which might in turn lead to the chronic systemic inflammation or destruction of the endothelial wall by the bacterium itself.^8,9^ This can be attributed to findings from previous studies which have shown the presence of some large periopathogens in the carotid artery atheromas.^10,11^ Bacteria that are closely related to the onset and progression of periodontal diseases such as *A. actinomycetemcomitans*(A.a), *Porphyromonas gingivalis* (P.g), *T. denticula*(T.d) are called periodontopathic bacteria.^12^

*Porphyromonas gingivalis*is a gram-negative, anaerobic bacterium which is observed in many periodontal diseases such as periodontal abscess, adult periodontitis and therapy-resistant periodontitis.^13^ It is often detected in deep dental pockets and is less prevalent in people with healthy periodontal conditions.^14,15^ This bacterium has filament protrusions on its surface called fimbria, which is a strong virulence factor of this bacterium in the onset and progression of the disease.^16^ The fimbria protein components facilitate the binding and accumulation of bacteria to the surface of the host's tissues, just as studies have shown its effect on bacterial adhesion to the salivary gland walls.^17,18^ According to nucleotide sequences, the genes of this bacterium (fimA gene) consist of 6 fimA genotypes, which can affect its pathogenesis.^19^ Evidence from previous studies showed that genotypes II and IV are more commonly seen in subjects with periodontal problems.^20,21^ Therefore, coupled with inadequate research among the Iranian population, the present study investigated the prevalence of *Porphyromonas gingivalis*fimA genotypes II and IV in the sub-gingival and atheromatous plaques in patients with chronic periodontitis and atherosclerosis.

## Materials and Methods

The present study was a cross-sectional observational study and approved by the Ethics Committee of Shahid Beheshti University of Medical Sciences, Tehran, Iran (Thesis number: 310131). The sample size was obtained based on the Cochran's sample size formula^22^ (15 subjects in each group) and the frequency considered in this formula was based on previous studies. A sample size of 15 each was collected for patients with periodontitis, atherosclerosis and patients with periodontitis + atherosclerosis. Samples were selected from the volunteers referred to Estakhr Clinic and Tehran Heart Center, Tehran, Iran and Sina Heart Center, Isfahan, Iran. Subjects with chronic moderate-to-severe periodontitis (as defined by Armitage)^23^ and pockets with a probing depth of at least 4 mm and CAL of at least 3 mm were assigned to groups A and C. Patients in group B had a healthy periodontal condition, i.e., the maximum probing depth of 3 mm and maximum CAL of 2 mm. Coronary artery bypass graft (CABG) patients were diagnosed by a cardiac surgeon. People who had a history of antibiotic use for the last 6 months, long-term use of immunosuppressive drugs, uncontrolled systemic diseases, immune deficiency, use of any tobacco products, periodontal treatment during the past 12 months, pregnant and lactating women, individuals with <12 teeth in their mouth and those with any contagious viral diseases such as HBV, HCV, HIV, etc. were excluded from the study.

### Sampling

In group A, the tooth sample was first isolated using cotton rolls and the supragingival plaque was removed from the tooth surface using sterile gauze. After determining the probing depth and identifying the deepest pockets in the mouth, the subgingival plaque specimen was removed from the 3 areas of the deepest pockets using a Gracey curette (Hu-Friedy, USA) (PD≥3 mm). While working with the curette, attempts were made to avoid damaging the soft tissue and gums and not to stain the plaque specimen with blood. The specimens were immediately placed in 1.5-mL microtubes containing lysis buffer (NaCl = 0.145 g/EDTA 0.5 M = 12.5 mL/Tris 0.5 M = 5 mL/SDS = 0.5 g) and the solution frozen in a freezer (-40°C). The gingival sulcus plaque and the aortic wall sampling were performed in group B. The gingival sulcus plaque sampling was performed similar to that in group A. To provide blood supply to the grafted vein during CABG surgery, the ascending aortic wall was pierced using a punch and one end of the vein was connected to the resulting hole. The surgeon later determined the location of the atheromatous plaque at the aortic wall during the surgery by a tactile sensation. In this study, the tissue isolated from the ascending aortic wall in the aortic punching stage was collected as the aortic wall specimen. This tissue was immediately immersed in 1.5-mL microtubes containing the lysis buffer, and the solution was frozen in a freezer at -40°C. In group C, the sampling of the aortic wall and the gingival sulcus plaque sampling were similar to that in previous groups.

### Laboratory procedures

Microtubes were transferred to a laboratory in the Molecular Cell Research Center, Faculty of Medicine, Shahid Beheshti University of Medical Sciences at 0°C. Using PCR technique and 16S rDNA primer, the presence or absence of P.g bacterium in the specimens was investigated in the laboratory. After retrieval from the lysis buffer, the bacterial gingival sulcus plaques underwent the extraction process without any extra process. However, the aortic wall specimens were divided into finely divided pieces after being removed from the lysis buffer with a surgical blade and then were subjected to the extraction process. DNA extraction was performed using tissue kit (Qiagen Mini, QIAamp, Germany). 16s rDNA primers as well as primers of genotypes II and IV were designed and developed by the NCBI Bank Gene and made by Sinaclon Co (Table 1).

**Table 1:** Sequence and melting temperature of primers

**Primer**	**Sequence**	**Melting temperature (ºC)**
**60s rDNA**	F 5′-AGG CAG CTT GCC ATA CTG CG-3′ R 5′-ACT GTT AGC AAC TAC CGA TGT-3′	60
**Genotype II**	F 5`-ACA ACTATA CTTATG ACA ATG G-3` R 5`-AAC CCC GCT CCC TGT ATT CCG A-3`	55
**Genotype IV**	F 5`-CTA TTC AGGTGC TAT TAC CCA A-3` R 5`-AACCCC GCT CCC TGT ATT CCG A-3`	52

The PCR reaction was performed using an Eppendorf gradient (Eppendorf, Germany) on a solution (1 μL of DNA, 2 μL of primer, 8 μL of Master mix (AMPLIQON, Denmark), and 9 μL of deionized water). Based on previous studies,^24^ the solution was initially placed in a cycle for 5 minutes at 95°C, followed by 30 cycles of 30 seconds at 94, 60 and 72°C, respectively. Finally, the last cycle was performed for 7 minutes at 72°C. After completing the PCR process, the resultant solution was mixed with dye solution (0.6% SDS + 60 Mm EDTA + 12% Glycerol + Syber + Chromophenol blue) and electrophoresed on 1.5% agarose gel. After electrophoresis, the gel was placed on a UV duct (UVTEC, USA) and the results were determined after observing the bands. Chi-squared and Fisher’s exact tests were used to determine the relationship between the prevalence of P.g and its genotypes with severity of chronic periodontitis and atherosclerosis. One-way ANOVA and independent t-test were also used to study the relationship between the prevalence of P.g and its genotypes with severity of atherosclerosis.

## Results

Based on the information provided by the collaborating centers of the project stating that the two specimens were infected with HCV virus and the non-Iranian nationality of one sample donor, a total of 3 gingival sulcus plaques were removed at the completion of the sampling step and at the start of the laboratory phase. Consequently, there were 15, 13 and 14 subjects in groups A, B and C, respectively. In this cross-sectional study, 45 subgingival plaque specimens and 30 aortic wall specimens were evaluated to determine the presence or absence of *P. gingivalis* and its fimA II and IV genotypes, by using the PCR technique. Fisher’s exact test results showed a significant difference between patients in groups B and C in terms of *P.gingivalis*prevalence rate in the gingival sulcus specimens at P=0.001 (odds ratio=0.03). However, there was no significant difference between patients in groups A and C in terms of the prevalence of moderate periodontitis (MP) and severe periodontitis (SP) (Table 2).

**Table 2:** Results of investigation of the prevalence of *P. gingivalis* 16s rDNA primer and PCR technique for gingival sulcus specimens in each group

**Group**	**Periodontal condition**		**DNA gingiva**		**Total**
			**+**	**-**	
**A (periodontitis)**	MP	Number	3	6	9
		Percentage	33.3%	66.7%	100%
	SP	Number	4	2	6
		Percentage	66.7%	33.3%	100%
**B (atherosclerosis)**	Healthy	Number	1	12	13
		Percentage	7.7%	92.3%	100%
**C (periodontitis + atherosclerosis)**	MP	Number	6	4	10
		Percentage	60%	40%	100%
	SP	Number	4	0	4
		Percentage	100%	0%	100%

There was no statistically significant difference between groups B and C in terms of the prevalence of *P.gingivalis*among patients at P=0.462 (Table 3).

**Table 3:** Results of investigation of the prevalence of P. gingivalis 16s rDNA primer and PCR technique for the aortic wall specimens in each group

**Group**	**Periodontal condition**		**DNA gingiva**		**Total**
			**+**	**-**	
**B (atherosclerosis)**	Healthy	Number	1	12	13
		Percentage	7.7%	92.3%	100%
**C (periodontitis + atherosclerosis)**	MP	Number	0	10	10
		Percentage	0%	100%	100%
	SP	Number	0	4	4
		Percentage	0%	100%	100%

In the gingival sulcus and aortic wall specimens, the results of PCR showed no *P.gingivalis*fimA II and IV genotypes. The results also revealed that the mean Gensini scores (atherosclerosis severity)^25^ in patients with healthy periodontium, moderate chronic periodontitis and severe chronic periodontitis were 46.19±19.93, 61.05±30.79 and 63.38±26.21, respectively. These results were not statistically significant at P=0.30. Mean Gensini scores of 22.82±48.78 and 28.31±62.18 were obtained for patients without and with *P.gingivalis*genotype in their gingival sulcus plaques, respectively. These results were not statistically significant at P=0.19 (Table 4).

Considering the fact that P.g genotype was recorded only in one specimen, it was impossible to carry out statistical tests to investigate this relationship.

**Table 4:** The results of gingival sulcus and aortic wall specimens PCR analysis

		**Number**	**Mean**	**Standard deviation**	**Minimum**	**Maximum**
**Periodontal status**	Healthy	13	46.19	19.93	26	100
	mp	10	61.05	30.79	18	118
	sp	4	63.37	26.21	48	102
**The bacterial prevalence rate in the gingival sulcus**	+	11	62.18	28.31		
	-	16	48.78	22.82		

## Discussion

Infections with oral origin have been considered as a cause of infections of heart tissues since 5 centuries ago.^26^ In 1988, the relationship between periodontitis and atherosclerosis was shown for the first time.^27^ Basic theories rely on epidemiological studies. Over time, the study of the relationship between cardiovascular and periodontal diseases entered a new phase with the advent of microbiological science and the introduction of new methods, such as PCR technique, for the identification of microorganisms. Although due to the nature of the disease and moral constraints, there has never been a study of the causal relationship between the two in human species, today many studies have shown the relationship between these two types of diseases.^28,29^ The two factors that are currently associated with both diseases are bacterial agents and immune responses. The present study investigated the prevalence of fimA II and IV genotypes in gingival sulcus and atherosclerosis plaques and their interaction with the severity of the disease. The results of the current study revealed that the P.g 16s rDNA primer was found in 46.7%, 7.7% and 71.4% of the gingival sulcus specimens obtained from groups A, B and C, respectively. Talebi et al^30^ conducted the only study on the same topic among the Iranian population under the same conditions. They reported a P.g prevalence rate of 61% in the gingival sulcus plaques of patients with chronic periodontitis. This difference is probably due to the different severity forms of the periodontitis studied because Talebi et al studied only the severe form of periodontitis, while the present study focused on moderate and severe forms placed in a group called patients with chronic periodontitis and the results showed that P.g-positive specimens were mostly affected by severe chronic periodontitis. Cairo et al^31^ (2004) reported a P.g prevalence rate of 90% in their study in the gingival sulcus plaques in patients with chronic periodontitis. Also, Aimeti et al^32^ reported a P.g prevalence rate of 63.3% in a study on the gingival sulcus plaques in patients with chronic periodontitis, while Morita et al^33^ (2014) reported a P.g prevalence rate of 62.5% in a study on subgingival plaques in patients with chronic periodontitis. These researchers used paper points for sampling gingival sulcus plaques, whereas curettes were used for sampling in the present study. Different methods of sampling and racial differences are among the possible causes of differences in the results. In this study, P.g II and IV genotypes were not detected in the gingival sulcus specimens in any groups. Additionally, there are various studies on the prevalence of different P.g genotypes in the gingival sulcus plaques of patients with chronic periodontitis in other countries but there are no consistent results as a whole. Previous studies carried out in Southeast Asia by Zhao et al^34^ (China) and Moon et al^35^ (South Korea) showed that genotypes II and IV of this bacterium recorded the highest prevalence in the subgingival plaques of patients with chronic periodontitis. However, previous studies performed on the gingival sulcus plaques in patients with chronic periodontitis in Spain by Puig-silla et al^36^ and in Brazil by Missailidis et al^37^ showed that II and IV were the most prevalent genotypes. In their independent studies on the gingival sulcus plaques in patients with chronic periodontitis, Nakano et al^38^ in Japan and Beikler et al^39^ in Germany showed that I and II were the most prevalent genotypes. Interestingly, Zao et al^34^ reported Type I genotype as the most common genotype in the gingival sulcus plaque in individuals with healthy periodontium.

Sterile curettes were used in Brazil and South Korea (as was also used in this study), while sterile paper points were used for sampling in Spain, Germany and China. Hence, the sampling method can be considered as a factor responsible for this difference in the results. However, Al Ahdab et al^40^ showed a statistically significant difference among the studies in terms of the amount of bacterial specimen taken by these two methods. Furthermore, the racial similarity between the Spanish and the Brazilian population seems to play a very important role in the prevalent genotype of this bacterium in the gingival sulcus plaques of patients with chronic periodontitis.

Results from different studies show that genotype II of this bacterium seems to be the most common genotype in the gingival sulcus specimens of patients with chronic periodontitis. Nevertheless, this genotype was not seen in any of the specimens in this study. It seems that both race and sampling method are factors involved in this difference. Our results showed that the P.g-16s rDNA primer was seen only in one aortic wall specimen (7.7%) in group B, while the P.g-16s rDNA primer was not observed in any of the specimens obtained from group C. The results of the present study are similar to those of three previous studies. In a study on the carotid arteries and aortic atheromatous plaques of 13 patients with different dental conditions, Fernandes et al^41^ (2014), using real time-PCR technique, reported a P.g prevalence rate of 0%. Aimeti et al^32^ (2007) investigated the carotid artery and the subgingival atheromatous plaques of 33 patients with chronic periodontitis using the PCR technique. Although these researchers found bacterial DNA in 93.94% of the atheromatous plaques, they reported a prevalence rate of 0% for DNA associated with oral pathogens, including P.g.^32^ In another study, Cairo et al^31^ (2004) divided patients with atherosclerosis into two groups of (N=26) edentulous and dentate individuals. Oral specimens and carotid artery atheromatous plaques were taken from each group and these researchers did not find any P.g genotype DNA in the atheromatous plaques.

The results of the study of aortic wall atheromatous plaques were inconsistent with the results of most studies on different vessels. For example, Talebi et al^30^ (2011) performed a study on 40 Iranian patients with atherosclerosis, and divided them into two groups of patients with and without chronic periodontitis. They later reported a P.g prevalence rate of 80% in the coronary artery atheromatous plaques. Mahendra et al^42^ (2014) investigated the coronary artery atheromatous plaques and gingival sulcus plaques of 51 patients with chronic periodontitis, who were candidates for CABG surgery. These researchers reported a P.g prevalence rate of 45.1% and 64.71% in the coronary artery atheromatous plaques and the gingival sulcus plaques, respectively. In a study on carotid artery atheromatous plaques of 42 Austrian patients, Figuero et al^43^ reported a P.g prevalence of 78.57% in these plaques. Morita et al^33^ (2013) investigated 16 cases of carotid artery atheromatous plaques of Japanese patients and reported a P.g prevalence of 62.5%. Pucar et al^44^ (2007) examined the coronary artery atheromatous plaques of 15 Serbian patients who were candidates for CABG surgery. These researchers reported a P.g prevalence rate of 53.33% in the specimens.

While reviewing the above studies, it is important to note the following points:

1. In these studies, atheromatous plaques were taken from coronary arteries or carotid arteries. Given the fact that atherosclerosis is a generalized disease in all blood vessels,^45^ and any manipulation or removal of atheromatous plaques in the coronary artery is contraindicated in the new protocols for CABG, in this study, aortic wall atheromatous plaques were taken. Also, according to a review article entitled “Aortic atherosclerotic disease and stroke”, which was published by Kronzon et al^45^ (2006), there is a correlation between the aortic calcification observed in chest radiography and the progression of coronary heart disease. In addition, it has been shown that the aortic plaque found in transesophageal echocardiography (TEE) is associated with a higher incidence of CAD and also a significant presence of stenosis in coronary artery angiography. Research has also shown that the lack of aortic plaques in the TEE can act as a predictor for the absence of CAD. In this study, a part of the aortic wall was removed by a punch during CABG surgery. Thus, use of a part of the atheromatous plaque as a specimen provides a chance to examine a larger segment of the plaque while using the part removed by the aortic punch as a specimen restricts this examination (due to containing part of the vessel wall and the availability of a smaller amount of plaque) despite the homogeneous nature of the atheroma plaque. This limitation of access to plaque mass in this study as compared to the above-mentioned studies can be considered as one of the possible reasons for the difference in the results obtained.
2. Studies have been conducted in different countries and on people of different races. As was earlier mentioned, race plays a decisive role in the spread of various bacterial species. This racial difference can be considered as one of the possible causes of the difference in results.
3. Exclusion criteria (such as smoking, diabetes and other systemic diseases) were not considered in the studies of Pucar et al^44^ and Morita et al,^33^ as these patients were included in the studies of these researchers. Although in these studies, attempts were made to adjust these individuals in groups, these conditions still have a strong effect on the diversity of microorganisms with normal flora and pathogens. In the present study, interventional factors were considered in the exclusion criteria and those with these factors were excluded from the study. The effects of cigarette and diabetes on the variation of microorganisms can be considered as one of the possible causes of the difference in results.
4. In the article of Cairo,^31^ entitled “Periodontal pathogens in the atheromatous plaques controlled clinical and laboratory trial A”, methodological differences were mentioned as one of the possible reasons for the difference in the results obtained (DNA extraction method, various primers and different PCR conditions used in different laboratories).
5. The groups in a study by Figuero^43^ used systemic antibiotics during sampling.

Finally, Cairo^31^ reported that another possible cause for the differences observed in the results might be the fact that the prevalence of periopathogenic bacteria in atheromatous lesions is affected by various epidemiological factors such as stage of disease, nutrition, and geographical and nationality factors. In this study, there was no significant relationship between Gensini score (severity of atherosclerosis) and the prevalence of P.g in the atheromatous plaques.

This finding can be explained by considering the following important points:

1.To detect P.g in the atheromatous plaques, their count must reach a certain level.

2.As previously mentioned, the sampling method used in this study evaluated a small amount of atheromatous plaque.

3.Both P.g II and IV genotypes were studied in the present study. It is likely that there is a relationship between Gensini score and other genotypes of this bacterium. There was a significant difference between patients with atherosclerosis in chronic periodontitis and normal periodontium cases (P<0.0001). However, there was no relationship between the frequency of this bacterium in the subgingival plaques of patients with normal and affected periodontium at OR=0.03.

## Conclusion

The results of this study showed no significant relationship between the prevalence of P.g II and IV genotypes in the subgingival plaques in the case of healthy periodontium, chronic periodontitis and the incidence and severity of atherosclerosis.

## Authors’ contributions

The study was planned by MRTA and AEN and MP. Data collection statistical analyses and interpretation of data was carried out by MRTA and AEN and BK and SKFN and MP and HM and MRHK. The manuscript was prepared by MRTA and AEN and MP and MRHK.

## Competing interests

No conflict of interest exists in relation to this study.

## Ethics approval

Not applicable.
